# A novel perfusion bioreactor promotes the expansion of pluripotent stem cells in a 3D-bioprinted tissue chamber

**DOI:** 10.1088/1758-5090/ad084a

**Published:** 2023-11-10

**Authors:** Elizabeth R Komosa, Wei-Han Lin, Bhushan Mahadik, Marisa S Bazzi, DeWayne Townsend, John P Fisher, Brenda M Ogle

**Affiliations:** 1 Department of Biomedical Engineering, University of Minnesota, Minneapolis, MN, United States of America; 2 Stem Cell Institute, University of Minnesota, Minneapolis, MN, United States of America; 3 NIBIB/NIH Center for Engineering Complex Tissues, College Park, MD, United States of America; 4 Fishell Department of Bioengineering, University of Maryland, College Park, MD, United States of America; 5 Department of Chemical Engineering and Materials Science, University of Minnesota, Minneapolis, MN, United States of America; 6 Department of Integrative Biology and Physiology, University of Minnesota, Minneapolis, MN, United States of America; 7 Lillehei Heart Institute, University of Minnesota, Minneapolis, MN, United States of America; 8 Department of Pediatrics, University of Minnesota, Minneapolis, MN, United States of America; 9 Institute for Engineering in Medicine, University of Minnesota, Minneapolis, MN, United States of America; 10 Masonic Cancer Center, University of Minnesota, Minneapolis, MN, United States of America

**Keywords:** 3D bioprinting, stem cells, bioreactor, extracellular matrix, proliferation

## Abstract

While the field of tissue engineering has progressed rapidly with the advent of 3D bioprinting and human induced pluripotent stem cells (hiPSCs), impact is limited by a lack of functional, thick tissues. One way around this limitation is to 3D bioprint tissues laden with hiPSCs. In this way, the iPSCs can proliferate to populate the thick tissue mass prior to parenchymal cell specification. Here we design a perfusion bioreactor for an hiPSC-laden, 3D-bioprinted chamber with the goal of proliferating the hiPSCs throughout the structure prior to differentiation to generate a thick tissue model. The bioreactor, fabricated with digital light projection, was optimized to perfuse the interior of the hydrogel chamber without leaks and to provide fluid flow around the exterior as well, maximizing nutrient delivery throughout the chamber wall. After 7 days of culture, we found that intermittent perfusion (15 s every 15 min) at 3 ml min^−1^ provides a 1.9-fold increase in the density of stem cell colonies in the engineered tissue relative to analogous chambers cultured under static conditions. We also observed a more uniform distribution of colonies within the tissue wall of perfused structures relative to static controls, reflecting a homogeneous distribution of nutrients from the culture media. hiPSCs remained pluripotent and proliferative with application of fluid flow, which generated wall shear stresses averaging ∼1.0 dyn cm^−2^. Overall, these promising outcomes following perfusion of a stem cell-laden hydrogel support the production of multiple tissue types with improved thickness, and therefore increased function and utility.

## 1. Introduction

Three-dimensional (3D) bioprinting offers vast potential for creating human models of disease, allowing *in vitro*, organ-level study of disease mechanisms, progression, and treatment. Bioprinting may also allow the generation of functional replacement tissues to serve as a therapy for various ailments. However, with billions of cells in each organ of the body, acquiring sufficient numbers of cells for creating representative *in vitro* tissues is a nontrivial task. While extrusion bioprinting typically allows for higher cell densities than other printing modalities, currently, it is typically only performed on the scale of tens of millions of cells per ml of bioink [1]. Methods are now being developed for scaffold-free bioprinting with spheroids or embryoid bodies [2–4], which allows for improved cell density in printed tissues. Yet, in addition to its own set of limitations, including challenges in spheroid variability, printability, and print resolution, these methods still require cell scale-up and large media volumes to achieve physiological densities.

To address the issue of low cell density in engineered tissues, the bioprinting approach of *in situ* stem cell expansion and differentiation has been introduced [5–11]. In this approach, stem cells, instead of parenchymal cells, are printed in the bioink. The stem cells can be expanded in the engineered tissue prior to differentiation to the desired cell type. This is especially beneficial for applications involving cell types with limited proliferative or migratory capacity, such as cardiomyocytes, or as an alternative to the use of patient-derived primary cells [5, 12, 13]. The other advantage of this approach is that critical cell–cell, cell-extracellular matrix (ECM) and ECM–ECM attachments formed with differentiation can be maintained without disruption. This is important, as ECM organization has been found critical for the function of engineered cardiac tissue [14]. Further, the microenvironments formed during *in situ* differentiation and preserved in a 3D-bioprinted structure enable the organization and cardiac function of a human chambered muscle pump (hChaMP) [5].

The hChaMP, printed with human induced pluripotent stem cells (hiPSCs), is generated from a bioink containing ECM optimized for hiPSC growth and differentiation to cardiomyocytes. hiPSCs proliferate for two weeks prior to cardiomyocyte differentiation, resulting in a more physiologic cell density in the structure. For this reason, the hChaMP demonstrates contiguous muscle function throughout the structure, as demonstrated by optical mapping and the capacity to pump liquid with each contraction of the muscularized tissue. Yet, while bioprinting with stem cells and subsequent expansion and differentiation is a promising technique, the hChaMP demonstrates limited tissue thickness due to poor nutrient availability [5], a well-known issue in the field of tissue engineering.

The availability of nutrients, particularly oxygen, limits the thickness of engineered tissues, typically reaching only 100–200 *μ*m of viable tissue [15]. However, with fluid flow, nutrient access can be expanded via convection, leading to the development of numerous bioreactors for engineered tissues [16, 17]. As pluripotent stem cells are sensitive to the microenvironment, and shear stress has been shown to induce hematopoiesis in mouse stem cells [18] and promote formation of endothelial cells from stem cell-derived precursors [19, 20], convective flow for hiPSC expansion in engineered tissues may induce unintentional differentiation. Conversely, many bioreactor studies have shown the promise of using hydrodynamics to expand stem cells as aggregates or with microcarriers with limited loss of potency [21–25]. Here, we describe the development of a perfusion bioreactor for the hChaMP. We show that intermittent perfusion of the hChaMP yields a nearly two-fold increase in cell density after 1 week of culture. Further, hiPSCs remain pluripotent despite exposure to shear stress, which was approximated to be lower than other common bioreactors for stem cell expansion. These results suggest promising potential for generating thick-tissue models upon differentiation of expanded iPSCs to desired cell types.

## 2. Materials and methods

### 2.1. hChaMP fabrication

**Figure 1.**
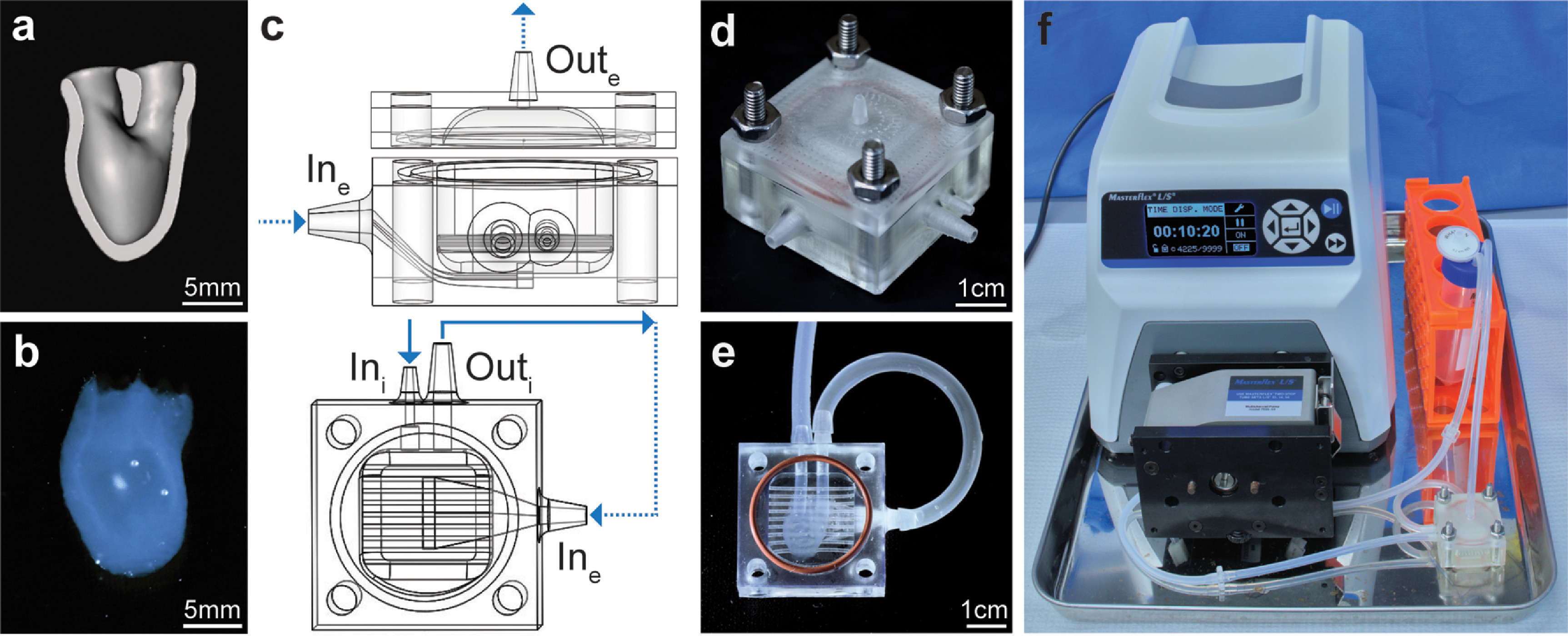
hChaMP and bioreactor design. (a) Cross-section (frontal plane) of the hChaMP model showing the interior chamber for perfusion. (b) 3D-bioprinted hChaMP. (c) Bioreactor model, side view of the bioreactor with the top and bottom portions (top) and top-down view of the bottom portion that holds the hChaMP (bottom). The design allows for perfusion of the hChaMP interior (solid arrows), as well as fluid flow around the hChaMP exterior (dashed arrows). The hChaMP lightly rests on the thin horizontal bars, allowing for exterior perfusion around the underside of the hChaMP. (d) 3D-printed bioreactor, assembled with nuts and bolts and a silicone gasket. (e) hChaMP in the bioreactor. Tubing connects the hChaMP to the bioreactor. (f) Entire perfusion set-up with bioreactor connected to a pump and media reservoir.

hChaMPs were redesigned from the previously-reported structure [5] via Autodesk Meshmixer and Blender (figure 1(a)), and a g-code was generated in Slic3r. hChaMPs were fabricated (figure 1(b)) as previously reported [5]. One day prior to printing, gelatin methacryloyl (GelMA; University of Minnesota Bioprinting Facility) was reconstituted to 26.7% w/v at 60 °C in a solution containing 75% mTeSR1 (STEMCELL Technologies, cat#85850), 25% acetic acid (20 mM), and 1.3% w/v lithium phenyl-2,4,6-trimethylbenzoylphosphinate (LAP, Allevi). Methacrylated collagen (ColMA; Advanced Biomatrix, cat#5198) was solubilized to 2% w/v in 20 *μ*M acetic acid at 37 °C. Prior to printing, the GelMA and ColMA solutions were mixed (3:1 GelMA solution:ColMA solution, giving 20% w/v GelMA, 0.5% w/v ColMA) and neutralized with 1 M sodium hydroxide.

hiPSCs (overexpressing cyclin D2 under the myosin heavy chain gene [26], female) were used in these studies. hiPSCs were maintained in mTeSR1 on Matrigel (Corning, cat#08-774-552) and were regularly passaged via ReLeSR (STEMCELL Technologies, cat#05872). For printing, hiPSCs at 60%–80% confluency were singularized with Accutase (Millipore Sigma, cat#A6964). The cell suspension was washed and centrifuged twice to remove any excess Accutase that may degrade the matrix-based scaffold. Cells were then resuspended at 30 million cells ml^−1^ in an ECM solution, containing 187 *μ*g ml^−1^ mouse laminin/entactin (LN; Corning, cat#354259), 187 *μ*g ml^−1^ human fibronectin (FN; Corning, cat#356008 or R&D systems, cat#1918-FN-02M), and 10 *μ*M Y-27632 ROCK inhibitor (Selleckchem, cat#S1049) in mTeSR1, and incubated at room temperature for 15 min. The cells in ECM were mixed 1:1 with the GelMA/ColMA mixture, giving the ink a final composition of 15 million cells ml^−1^ in 10% w/v GelMA, 0.25% w/v ColMA, 93.75 *μ*g ml^−1^ LN and FN, 0.5% w/v LAP, and 5 *μ*M Y-27632. hChaMPs were printed on an INKREDIBLE extrusion bioprinter (CELLINK). For high resolution with our low-viscosity bioink, printing was conducted in a support bath based on FRESH v2.0 [27]. The support bath was created in a biological safety cabinet to maintain sterility; a mixture of 52.5% sterile Milli-Q water and 47.5% ethanol was heated to 45 °C in a water bath created in a secondary container. The mixture was stirred at 400 rpm and covered with foil to minimize evaporation. Sodium hydroxide (1 mM) was added to raise the pH to 7, followed by addition of gelatin type B (Sigma Aldrich, cat#G9382, to 2% w/v) and gum arabic (Sigma Aldrich, cat#9752, to 1% w/v). The solution was mixed at 45 °C for 10 min before being cooled to 35 °C, followed by slow cooling to 25 °C (<1 °C min^−1^). Temperature was then lowered to 10 °C and stirred for an additional 5 min. The mixture was transferred to conical tubes and centrifuged at 100 G for 4 min. Supernatant was replaced with cold, sterile Milli-Q water, the tubes were vortexed and centrifuged at 500 G for 5 min. The product was then diluted in cold, sterile 1X phosphate-buffered saline (PBS; Fisher Scientific, cat#BP3994), and stored at 4 °C until the day of printing. At this time, the bath was centrifuged at 1200 G for 3 min to remove excess PBS and UV irradiated in thin layers for 30 min.

The bioink was printed at 27 °C with a 27 G, 1-inch needle. For consistent print structures, important for the perfusion set-up, pressure was varied based on the drop rate of ink from the needle, as previously described [5]. With the ink used here for the given print structure, a drop rate of 3.1 s between drops from the needle was applied. Ink was crosslinked with blue light (405 nm) after printing for 20 s on each of six faces. The prints in the support bath were then moved to an incubator to melt the support bath, followed by three washes with warm, sterile Dulbecco’s PBS (DPBS; without Ca^2+^ and Mg^2+^; Thermo Fisher Scientific, cat#14190144). hChaMPs were cultured overnight in mTeSR1 in 5 *μ*M Y-27632, under static conditions to allow for cell attachment.

### 2.2. hChaMP perfusion

Bioreactors were designed in SOLIDWORKS with ports for interior hChaMP perfusion (In_i_ and Out_i_) and ports for flow around the exterior of the hChaMP (In_e_ and Out_e_) (figure 1(c)). Bioreactors were 3D-printed (figure 1(d)) with E-shell 300 (EnvisionTEC, cat#1500300) via a Perfactory IV digital light projector printer (EnvisionTEC).

For perfusion, a Masterflex L/S Standard Digital Drive (cat#07522-30) was used with Masterflex L/S 14 platinum-cured silicone tubing (1.6 mm ID, cat#96410-14). The media reservoir was a 50 ml conical tube with three holes in the lid, two for the inlet and outlet of flow and one for a 0.22 *μ*m syringe filter (Whatman, cat#6780-2502) that allowed for sterile oxygen exchange in the media. The media reservoir was initially filled with double the volume of media (mTeSR Plus [STEMCELL Technologies, cat#100-0276] supplemented with 100 U ml^−1^ penicillin, 100 ug ml^−1^ streptomycin [pen-strep; Thermo Fisher Scientific, cat#15140122] for the first day of perfusion) than the system required in the tubing and bioreactor. This allowed for 50% media changes (mTeSR Plus, no pen-strep) on subsequent days by a simple swap of the conical tube to minimize the possibility of contamination and bubble formation that could potentially occur when draining the tubing. The static control was cultured in an upright T25 flask with an equivalent volume of media, also receiving 50% media changes daily.

Perfusion began one day after printing. Bioreactors were sterilized with a 30 min soak in 70% ethanol under UV light in a biological safety cabinet. Bioreactors were then washed three times with sterile 1X PBS. To prepare the hChaMP for perfusion (figure S1), the hChaMP was moved to a Petri dish with warm DPBS. Silicone tubing (15 mm long, 2 mm ID, 3 mm OD; uxcell, cat#a19011600ux0295) was inserted into both vessels of the hChaMP. The hChaMP with inserted tubing was moved to a clean, dry Petri dish, and sterile 20% GelMA (with 0.5% w/v LAP, dissolved in mTeSR1 and acetic acid as described above) was pipetted at the interface of the tubing and hChaMP while crosslinking with blue light. The hChaMP was moved to the bioreactor filled with DPBS and the other end of the tubing was inserted into the bioreactor channels using sterile, fine-tooth forceps (figure 1(e)). The 20% GelMA was added to both ends of each tubing to create a seal against leakage. The tubing for Out_i_ (Masterflex C-Flex tubing, 3.2 mm ID, cat#06422-04) was filled with DPBS and attached to the bioreactor to remove surface tension at Out_i_ which creates pressure differentials that hinder perfusion through the hChaMP. To check for leaks in internal perfusion, DPBS dyed with food coloring (filtered through a 0.22 *μ*m filter) was injected at In_i_ with a syringe and needle. More 20% GelMA was applied if leaks existed at the tubing connections. Dyed DPBS was washed out with warm mTeSR Plus. The bioreactor was then attached to the perfusion set-up.

With the bioreactor attached to the primed tubing, a 30 mm silicone gasket was placed on the bioreactor and the lid of the bioreactor was attached with nuts and bolts. The pump was turned on to 3 ml min^−1^ to remove remaining air for the bioreactor. The perfusion set-up (figure 1(f)) was moved to an incubator (37 °C, 5% CO_2_), and flow was changed to intermittent, 15 s of flow at 3 ml min^−1^ every 15 min. The 50% media changes were performed daily until the end of perfusion.

### 2.3. Computational simulations

To create a computational model of fluid flow in the hChaMP, pressure under flow was measured at the inlet and outlet of the bioreactor. Briefly, an acellular hChaMP was inserted in the bioreactor and attached for flow, as described above. A hemostasis valve (Qosina, cat#80395) was placed at the base of the bioreactor inlet or outlet to allow insertion of a pressure catheter in the set-up. Pressure profiles were measured with an ADV500 PV system and a 1.4 Fr pressure catheter (Transonic) with data acquisition at a sampling rate of 5000 Hz.

The interior flow was resolved using fluid–solid-interaction (FSI) approach in Simvascular [28]. The wall was described using a neo-Hookean model with an elastic modulus of 1.96 kPa, per experimental results. The fluid was modeled as Newtonian with a viscosity of 1 cP. A waveform was used to describe the intermittent flow in the inlet, and a pressure matching the experimental results was imposed as boundary condition at the outlet. Simulations were run on the Minnesota Supercomputing Institute for ten cycles in order to stabilize the pressure in the system.

The exterior flow was resolved using Ansys Fluent, release 20.1, assuming rigid walls. Like the interior flow simulations, the fluid was described as Newtonian fluid with a viscosity of 1 cP, and a waveform was imposed as the inlet boundary condition, with the flow rate matching the outlet results from the interior flow simulations. The outlet was set as the zero-reference pressure.

### 2.4. Cryosectioning and immunofluorescence (IF) staining

After 7 d of culture, the bottom of the hChaMP (the apex) was removed for ribonucleic acid (RNA) extraction (see below). The remainder of the hChaMP was fixed overnight in 4% paraformaldehyde at 4 °C. The following day, hChaMPs were washed three times with 1X PBS. The hChaMP chamber (between the vessels and the removed apex) was cut into two portions to allow for sectioning from different regions of the structure. Samples were incubated in 30% w/v sucrose in PBS for 2 d at 4 °C prior to sectioning. To prepare the samples for freezing, samples were moved to a 1:1 mixture of 30% sucrose:O.C.T. Compound (Tissue-Tek, cat#25608-930) for 1.5 h, followed by a 3 h incubation in O.C.T. Compound in cryomolds. The samples were then frozen at −80 °C.

Samples were sectioned at a thickness of 10 *μ*m using a Leica CM1900 cryostat. Slides were left at room temperature overnight and used or moved to −20 °C the following day. To image cell distribution through the hChaMP, sections were stained with 5 *μ*g ml^−1^ 4′,6-diamidino-2-phenylindole (DAPI, Invitrogen, cat#D1306) for 8 min and mounted with a solution of DAPI/1,4-diazabicyclo [2,2,2] octane (DABCO; Sigma-Aldrich, cat#D27802) in glycerol and PBS. Slides were imaged as tilescans on a Leica DMi8 fluorescence microscope. To quantify colony density in ImageJ, edges of sections were traced using tilescan brightfield images, DAPI-labeled tilescans were made binary, and the Analyze Particles function was used to identify cell colonies ⩾1000 *μ*m^2^. Five sections were analyzed per hChaMP. Distribution was quantified by drawing ten lines across the thickness (from outside to inside of chamber) of each section on brightfield tilescans (where colonies could not be easily visualized) and then finding the Plot Profile of the binarized DAPI along each line. Profiles were analyzed in Microsoft Excel to determine occurrence within each fifth section across the tissue thickness. The average wall thickness was calculated as the average length of all lines.

For IF, slides were permeabilized with 0.2% Triton X-100 (Sigma-Aldrich, cat#T8787) for 1 h and blocked with a solution of 5% bovine serum albumin, 1% glycine, 2% goat serum, and 0.1% Triton X-100 (BGST) for 2 h. Primary antibodies (1 *μ*g ml^−1^ mouse anti-OCT3/4 [Santa Cruz Biotechnology, cat#sc-5279], 5 *μ*g ml^−1^ mouse anti-KI67 [BD Biosciences, cat#556003], and 2.5 *μ*g ml^−1^ rabbit anti-PAX6 [Invitrogen, cat#42-6600], figure S2) in BGST were incubated on the sections overnight at 4 °C. Sections were then washed twice with 0.2% Tween-20 (Millipore, cat#655204) in 1X PBS and once with 1X PBS before 2 h incubation in secondary antibody (4 *μ*g ml^−1^ AlexaFluor 647 goat anti-mouse [Invitrogen, cat#A21236] or AlexaFluor 647 goat anti-rabbit [Invitrogen, cat#A32733]). Samples were again washed with Tween-20 and PBS prior to being stained with DAPI and mounted with DAPI/DABCO as described above. The 20× images of colonies were acquired and the area of fluorescent markers was quantified in ImageJ. Specifically, DAPI images were made binary, and the Analyze Particles feature was used to trace colony borders as regions of interest (ROIs) for colonies ⩾1000 *μ*m^2^. Then, the corresponding IF image was made binary, and the area covered by the marker within each ROI was quantified and normalized to DAPI density in the corresponding ROI. For each marker, >30 colonies were analyzed per hChaMP.

### 2.5. RT-qPCR

The apex of the hChaMPs were gently removed with a razor blade and flash froze in a liquid nitrogen tank for future RNA extraction. Samples were lysed and RNA isolated using the PureLink RNA Mini Kit (Invitrogen, cat#12183018A). Quality and quantity of RNA were quantified on a Cytation3 imaging reader (BioTek) using Gen5 Software. cDNA was created with the SuperScript IV VILO Master Mix (Invitrogen, cat#11756050), according to manufacturer’s instructions. Reverse transcription-quantitative polymerase chain reaction (RT-qPCR) was performed with *Power*SYBR Green Master Mix (1X, Applied Biosystems, cat#4367659) on a Roche LightCycler 96 (pluripotency markers, 60 °C annealing) or Eppendorf Mastercycler ep *realplex* ^2^ (germ layer markers, 55 °C annealing) for 30 cycles. Expression of the markers were normalized to GAPDH. Primer sequences (Integrated DNA Technologies, table S1) were found from literature, and primer pairs were chosen based on specificity for the gene of interest in primer-BLAST [29] and low possibility of dimerization according to Integrated DNA Technologies’ OligoAnalyzer Tool. hiPSC-derived germ layer tissues for positive controls were derived as described in the supplemental information.

### 2.6. Statistics

Three hChaMPs per condition were tested. Sample group comparisons were analyzed in JMP Pro 16.0. *P*-values were calculated for a null hypothesis of unequal means (two-tailed) with a 95% confidence interval. Student’s *t*-tests were used to calculate the *p*-values comparing static and perfused hChaMPs, whereas the multiple conditions from RT-qPCR were compared with analysis of variance (ANOVA) tests and post-hoc Tukey–Kramer honestly significant difference (HSD) tests.

## 3. Results

### 3.1. Bioreactor design and selection of perfusion parameters

The hChaMP structure designed for perfusion is shown in figure 1(a) (video S1). The structure was modified from the previously reported structure [5] to act as a single cardiac ventricle, allowing for future modeling of ventricular function (i.e. pumping) with applied fluid flow and mechanical valve actuation. The hChaMP, bioprinted via extrusion printing (figure 1(b)), is formed from a bioink containing hiPSCs at 15 million cells ml^−1^, gelatin methacryloyl (GelMA), methacrylated collagen, laminin/entactin, and fibronectin.

Due to the delicate nature of the ECM-based scaffold, with a storage modulus of 1.79 ± 0.13 kPa (figure S3), a bioreactor was designed to support the hChaMP throughout perfusion (figures 1(c)–(e)). Short pieces of tubing are used as a connection between the hChaMP and the bioreactor. The tubing is inserted into the hChaMP and sterile 20% GelMA is added and photocrosslinked at the connection to seal the tubing in place. The bioreactor contains a compartment to stably position the hChaMP, with two channels for insertion of the tubing attached to the hChaMP. The bioreactor was designed to properly guide fluid through the interior of the hChaMP and then around the exterior (figure 1(c) and videos S2–S3) to maximize nutrient exchange. To reduce fluid resistance through the hChaMP, Out_i_ was made larger than In_i_. The flow from Out_i_ leads to the In_e_, which enters the bioreactor compartment below the hChaMP and exits Out_e_ from the lid above the hChaMP (figure 1(c) and video S3).

**Figure 2.**
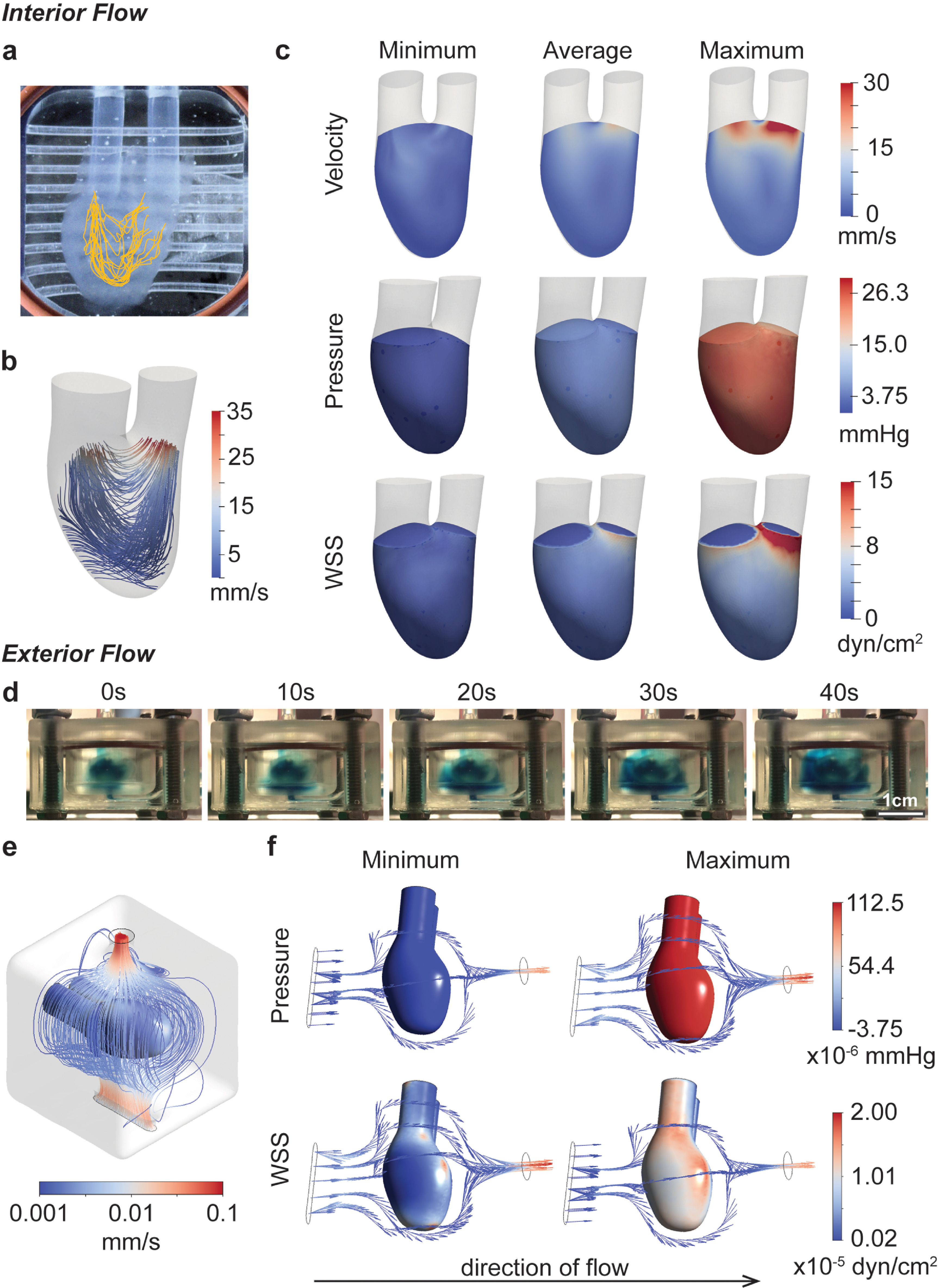
Fluid flow characterization. (a), (b) Flow patterns through the hChaMP chamber are summarized through particle tracking (a) and computational fluid dynamics (b). (c) Computational models provided estimates for the impact of perfusion on velocity, pressure, and wall shear stress on the interior hChaMP wall. (d) Summary of flow around the exterior of the hChaMP, demonstrated using DPBS dyed with blue food coloring. Fluid flow around the exterior is slow relative to flow through the interior of the hChaMP. (e) Computational model of flow profiles around the exterior of the hChaMP. (f) Computationally-approximated values of pressure and wall shear stress caused by exterior fluid flow (with the direction of flow shown here from left to right).

Upon completion of a bioreactor design that provided both interior and exterior flow, flow rates for perfusion were considered. Flow rates were optimized to minimize the shear stress experienced by cells in the hChaMP while also providing adequate nutrients throughout the entirety of the chamber. A flow rate of 3 ml min^−1^ was used, as rates below this did not allow fluid to reach the bottom of the chamber before exiting the chamber, creating dead zones of no flow (figures 2(a) and S4). However, continuous flow at this rate or higher damaged the hydrogel; thus, intermittent perfusion was tested. Infrequent perfusion, 1 min every 30–60 min, caused little cell growth, presumably due to the lack of oxygen exchange within the bioreactor and hChaMP and limited waste removal. Therefore, intermittent perfusion at 15 s every 15 min was employed. This duration of flow allowed for full media changes within the interior of the hChaMP, and the frequency supported cell survival within the tissue.

Of note, in cases where increased pressure was applied to the hChaMP in the bioreactor, leakage would consistently occur at the tubing-hChaMP interface. This pressure limitation restricts the utility of the model for downstream applications, as the hChaMP is not able to reach higher pressures when there are leaks at the interface. Therefore, methods were explored to improve the adherence of the soft tissue to the hydrophobic tubing. With pretreatment of 3-(Trimethoxysilyl)propyl methacrylate (supplemental methods, figure S5(a)), the tubing showed improved bonding to GelMA added at the tubing-hChaMP interface. This allowed the interface to withstand higher pressures, and the hChaMP itself was found to reach a maximum pressure of 16.8 ± 0.6 mmHg before rupture (figures S5(b), (c) and video S4). This is double the average pressure the system experiences with the peristaltic pump (∼8.2 mmHg). However, this maximum, or ‘burst’, pressure was found to be lower than the maximum pressure applied by the peristaltic pump (∼27.1 mmHg). This discrepancy is attributable to the oscillatory pressures of the peristaltic pump, where rapid fluctuations of low and high pressures cause expansion and contraction of the hChaMP (video S3). With the burst test, pressure was slowly applied directly to hChaMP walls, causing continuous expansion of the structure (figure S5(b) and video S4) until the wall became too weak to hold more fluid.

### 3.2. Approximations of fluid flow parameters

To approximate the effects of fluid flow on the hChaMP, particularly pressure and shear stress at the hChaMP walls, computational simulations were generated. Pressure profiles were measured at the hChaMP inlet and outlet using a pressure catheter inserted into a hemostasis valve at the base of In_i_ or Out_i_. Measured maximum, minimum, and average of the pressure profiles were used as the input for hChaMP flow in the computational model. SimVascular svFSI solver, an open-source software tailored for cardiovascular simulations [28], was used to solve for the internal flow profiles in the hChaMP. The software has been previously validated against *in vitro* models and has demonstrated its effectiveness in describing flow dynamics [30]. With this software and the 3D model of the hChaMP, fluid flow profiles matched those found from the *in vitro* experiment (figures 2(b), S4 and video S2), confirming the ability of the model to replicate the system. This allowed for computation of the generated shear stress on the inner wall of the hChaMP, a critical parameter in stem cell culture.

Pressures within the hChaMP were found to be fairly uniform, where variations throughout a single pump cycle matched experimental values, with an average around 4.5–5.3 mmHg and a maximum of 25.5 mmHg (figure 2(c)). Pressure was negative at its minimum, reflecting what is seen during the pulsatile cycling of the pump. This variability in pressure did not appear to cause issues on the integrity of the hChaMP after several days of perfusion. Wall shear stress was less variable, with an average value around 1.0 dyn cm^−2^, and a maximum of 2.7 dyn cm^−2^ through most of the hChaMP chamber, although drastically increasing near Out_i_ as fluid exited the chamber, reaching up to 15.0 dyn cm^−2^ (figure 2(c)).

Owing to the intricate geometries present in the external flow, Ansys Fluent was used to solve for the external flow profiles. Fluent serves as a commercial alternative to the open-source SimVascular and has been extensively utilized to demonstrate fluid flow patterns in the cardiovascular system, including *in vitro* models [31]. Solving for the external flow profiles enabled computation of wall shear stress on the exterior wall of the hChaMP. Pressures and wall shear stresses were much lower on the exterior, likely due to the large compartment size of the bioreactor and a pressure drop from Out_i_ to In_e_, leading to lower flow rates around the hChaMP (figures 2(d)–(f)).

Interestingly, pressure on the exterior depended on the location analyzed. The underside, where the fluid first contacts the hChaMP, had a higher maximum pressure than the upper side; yet the difference is fairly small, with a maximum pressure 1.13 × 10^−4^ mmHg for the underside and 1.05 × 10^−4^ mmHg for the upper portion. Similar to the pressures found for interior flow, the minimum pressure on the exterior of the hChaMP is slightly negative, but on the magnitude of 10^−6^ mmHg. Wall shear stress was also much lower than on the interior, ranging from values on the order of 10^−7^–10^−5^ dyn cm^−2^ (figure 2(f)). Overall, interior wall shear stresses were found to be in a similar range as, and exterior shear stresses much lower than, values found in common bioreactors for stem cell expansion, which have been reported around 0.7–2.5 dyn cm^−2^ [32–34]. Moreover, stem cells embedded in hydrogels were found able to withstand stresses greater than 30 dyn cm^−2^ [22], suggesting the shear stresses approximated for our set-up are reasonable for hiPSC expansion.

### 3.3. Effect of perfusion on hiPSCs growth and distribution

**Figure 3.**
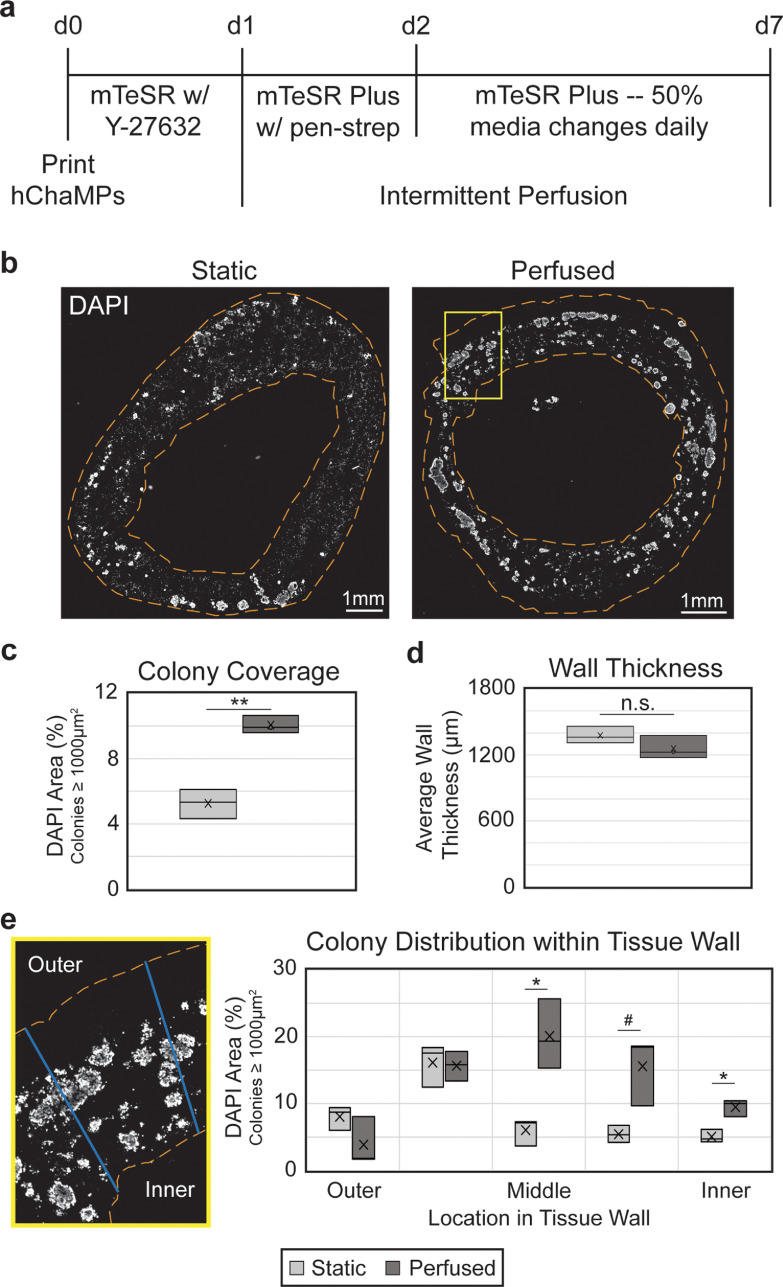
Perfusion improves hiPSC colony coverage and distribution. (a) Timeline of hChaMP proliferation, with intermittent perfusion beginning one day after printing. (b) hChaMP cross-sections, stained with DAPI (white) to show cell density throughout the hChaMP after 7 d of culture, with or without perfusion. (c) Colony density in the hChaMP is doubled in the perfused samples relative to static controls. (d) Wall thickness is not affected by intermittent perfusion. (e) Lines were drawn across hChaMP walls and processed in ImageJ to determine the distribution of colonies throughout the wall. Perfused hChaMPs had a large increase in colonies in the middle sections of the wall relative to static hChaMPs. Comparisons were performed between static and perfused hChaMPs at each region in the tissue wall. *n* = 3 hChaMPs. (n.s. not statistically significant; #*p* < 0.1, *p < 0.05, **p < 0.01).

To determine the effect of fluid flow on hiPSC proliferation and distribution, hChaMPs were inserted into the bioreactor for perfusion 1 d following printing, allowing time for cell attachment to the structure (figure 3(a)). Each subsequent day, conical tubes serving as the media reservoir were replaced with fresh media. Media in the tubing, corresponding to roughly half of the total volume in the system, was not drained to prevent the introduction of contamination or air bubbles into the system. Thus, a 50% media change with mTeSR Plus was performed daily. As biofabrication techniques in tissue engineering become more complex, the risk of contamination increases. Yet, the use of antibiotics on stem cells has generally been avoided, even for short-term treatment [35]. Here, penicillin-streptomycin was added on the first day of perfusion and gradually diluted with subsequent media changes. In this context, short-term exposure to penicillin-streptomycin had no impact on proliferation and potency of singularized hiPSCs in 3D culture (figure S6).

hChaMPs were cultured under flow for 6 d, for a total culture time of 7 d post-printing, before being fixed and cryosectioned for analysis. DAPI staining (figure 3(b)) was used to determine the size and location of viable cell colonies. In previous work (figure S7) we found that cells that were not part of colonies were not viable, as they had likely died during singularization required for fabrication and remained entrapped in the engineered tissue. Based on these criteria, we found the perfused structures exhibited a 1.9-fold increase in hiPSC colony density relative to controls in static media (figure 3(c)). Sectioned tissues were examined to verify there was no effect of flow on hChaMP integrity. Static and perfused hChaMPs maintained consistent wall thickness, around 1.2–1.4 mm (figure 3(d)).

Moreover, the distribution of hiPSC colonies within the walls was improved in perfused hChaMPs relative to hChaMPs in static cultures (figure 3(e)). The majority of hiPSCs in the perfused hChaMPs were located in the middle of the tissue wall, with less variation in the number of colonies among the different regions of the wall. On the other hand, the static control had more colonies located near the outer portion of the hChaMP, with lower distribution throughout the tissue. Interestingly, both perfused and static hChaMPs had a lower percentage of colonies in the outermost portion of the hChaMP relative to the next inner portion. This is possibly due to cell loss during support bath removal and media changes or perfusion.

### 3.4. Effect of perfusion on hiPSC pluripotency

**Figure 4.**
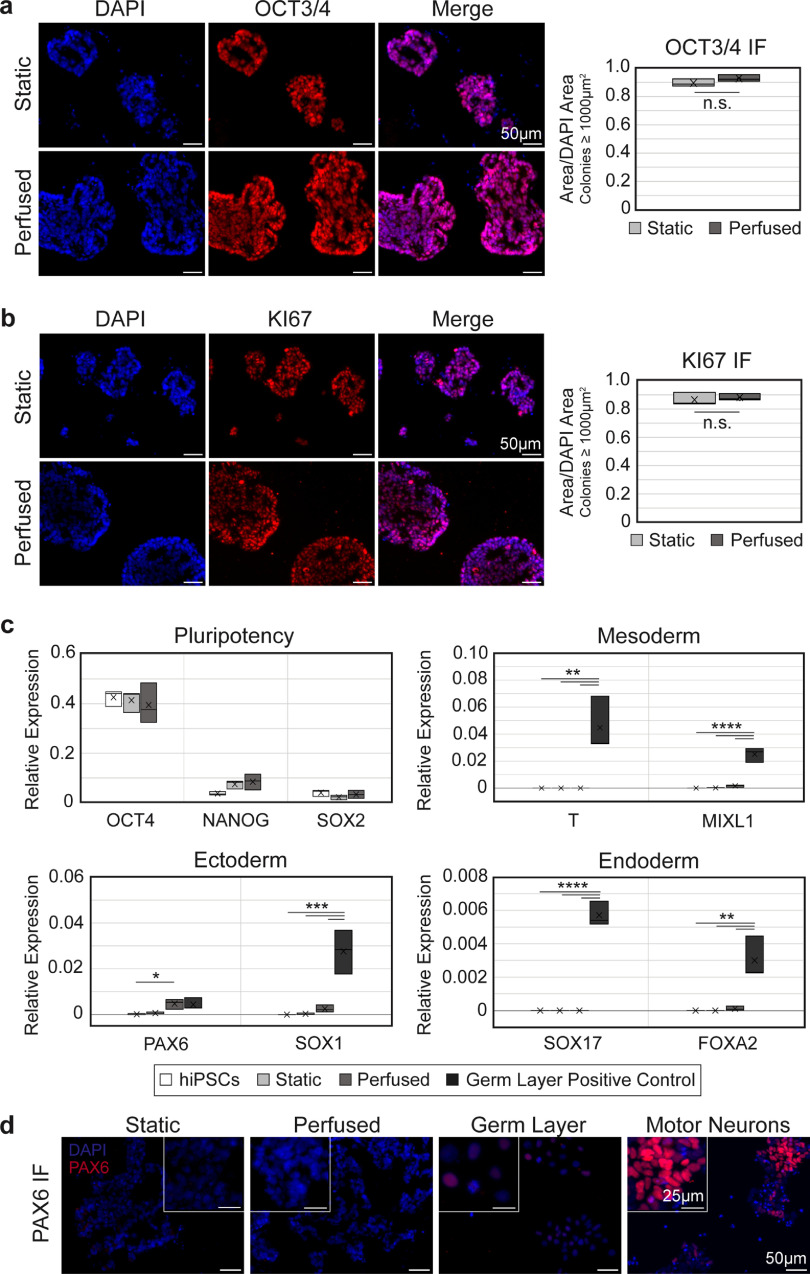
hiPSCs remain pluripotent and proliferative with perfusion. (a), (b) Expression of OCT3/4 (a) and KI67 (b) remains high in perfused hChaMPs, comparable to cells in the static control. *n* = 3 hChaMPs, >30 colonies per hChaMP. (c) RT-qPCR confirms a high expression of pluripotency markers OCT4, NANOG, and SOX2 and low expression of germ layer markers compared to negative (hiPSC) and positive controls (hiPSC-derived mesoderm, endoderm, or ectoderm), relative to expression of GAPDH. (d) Protein expression of PAX6 was not found in static or perfused hChaMPs but was found at low levels in the germ layer positive control. Motor neurons (day 12 of differentiation) are shown as a reference for high levels of fluorescent signal corresponding to high PAX6 expression. (n.s., not significant; *p < 0.05, **p < 0.01, ***p ⩽ 0.001, ****p < 0.0001).

To test whether shear stress from fluid flow caused undesired differentiation of the hiPSCs, IF staining for OCT3/4 was performed on cryosections of hChaMPs (figure 4(a)). Qualitative and quantitative analysis of colonies showed nearly all cells remained pluripotent, and pluripotency was comparable to the static control. Moreover, cells were highly proliferative, with positive expression of KI67 throughout the colonies (figure 4(b)). This suggests that hiPSCs retained their pluripotent and self-renewing characteristics upon application of intermittent perfusion.

Next, RT-qPCR was used to quantify pluripotency markers, as well as markers from each germ layer, to confirm our results on the transcript level (figure 4(c)). Expression of OCT4, NANOG, and SOX2 remained high in both static and perfused relative to the hiPSC control from 2D culture. Overall, germ layer markers were not detected, or were found at non-significant levels relative to hiPSCs or static hChaMPs, with one exception. Ectoderm marker PAX6 was found to be higher in cells from the perfused hChaMP relative to 2D hiPSCs (p=0.044). However, IF staining showed PAX6 was not expressed at the protein level in the hChaMPs (figure 4(d)).

## 4. Discussion

Here we demonstrate the ability to perfuse an hiPSC-laden bioprinted tissue with the goal of increasing the viable cell thickness of an engineered tissue. Perfusion provides a significant improvement of colony density after only 7 d of culture, half of our usual hChaMP proliferation period. Relative to previous perfusion bioreactor studies [36–40] that found improved viability within tissues using primary cell types, we were able to replicate the results using pluripotent stem cells, which are highly sensitive to their environment. Moreover, these previous studies typically perfused tissues interstitially, directly through pores of the scaffolds. Here, we demonstrate the ability to obtain similar results with bulk fluid flow around the tissue rather than directly through the tissue. Thus, the concepts described here associated with optimal bioreactor design, perfusion through the chamber of a delicate hydrogel, and flow considerations for hiPSCs will pave the way for studies of potentially any tissue type. Upon differentiation of this structure, thicker tissues can be generated, with an accompanying increase in tissue function.

Development of the bioreactor was an iterative process, where the most important parameters for successful proliferation were found to be delicate handling of the soft hydrogel and leak prevention at the connections to ensure perfusion through the hChaMP interior. Early attempts to perfuse the hChaMP in a vertical, upright position were successful in achieving increased cell viability on the interior. However, difficulty setting up the hChaMP for perfusion and a small compartment size required to hold the hChaMP in place, minimizing the volume of nutrients around the hChaMP, rendered these bioreactor designs impractical. Thus, the current bioreactor design positions the hChaMP in a supine position, providing easy set-up and sufficient media volumes for nutrient exchange. Delicate handling continues to be crucial to the success of perfusion though. In situations where hChaMP print resolution was poor, tubing insertion became more difficult and required harsher handling, causing drastically reduced colony formation by day 7 of culture. Further, pressure differentials played an important role in ensuring perfusion through the interior of the hChaMP. A common problem early in the design stage was leakiness at hChaMP tubing connections, despite addition of GelMA to create a seal. We found that before perfusion, tubing filled with DPBS must be attached at Out_i_ to remove surface tension, which created a pressure differential that hindered perfusion through the hChaMP interior, leading to leaks at the connections. This, combined with an increase in the leur size of Out_i_ relative to the inlet tubing, inherently removed this issue, promoting fluid flow through the interior of the hChaMP.

A large limitation to translational bioprinting methods is the need for massive amounts of cells to reach physiological densities, which is especially important as the size of engineered tissues increases to physiological scales. Allowing the cells to proliferate *in situ* reduces the need for large numbers of well plates and corresponding media volumes with monolayer expansion. Here, only one 6-well plate (10 million hiPSCs) is necessary to generate the centimeter-scale hChaMP. Further optimization of culture time, cell density at printing, or adjusted treatment with ROCK inhibitor and/or similar biochemical compounds could further increase the hiPSC density prior to differentiation.

Increased culture time would likely allow further cell growth but is undesirable for scale-up and tissue applications. This may also potentially further impact gene expression. While few changes in pluripotency or germ layer markers were seen at the RNA level by day 7 of culture, there was a slight increase in ectoderm marker PAX6 in perfused hChaMPs. At the protein level, though, PAX6 was not expressed in the hChaMPs. This outcome is consistent with studies of mouse PSCs exposed to shear stresses of 0.003–5 dyn cm^−2^, wherein expression of neural markers was comparable to static cultures [41, 42]. A slight, but non-significant, increase in NANOG RNA expression was also seen in perfused hChaMPs relative to hiPSCs cultured in 2D. Interestingly, a recent study found a similar increase in NANOG and OCT4 expression in mouse iPSCs exposed to flow, likely due to mechanotransduction involving E-cadherin and/or *β*-catenin [43]. Further, in human pluripotent stem cells, NANOG has been suggested to repress formation of ectoderm [44] and to increase cell proliferation by regulating entrance into the S-phase of the cell cycle [45], suggesting NANOG may play a beneficial role in the perfused hChaMPs. Thus, additional studies are necessary to determine the impact of prolonged perfusion on hiPSCs.

Cell density within the bioink could possibly be further optimized to increase colony numbers, but as discussed, is limited by nozzle sizes used for printing. Alternatively, treatment with 10 *μ*M ROCK inhibitors Y-27632 or Fasudil for multiple days was shown to enhance the rate of stem cell proliferation, with an increase in the percentage of cells in the S-phase [46]. Similarly, the flavonoid 3,2ʹ-dihydroxyflavone (3,2ʹ-DHF) has been shown to increase the proliferation of hiPSCs, the percentage of cells in S-phase, and expression of intracellular glutathione [47], which may play a role in epigenetic regulation of cell proliferation [48]. Combined treatment of hiPSCs with 3,2ʹ-DHF and Y-27632 further improved proliferation compared to either molecule alone [47], suggesting promising methods that could be coupled with perfusion to further improve cell density and tissue thickness.

With the desired hiPSC density in the hChaMP, the next step will be differentiation. Previous studies have shown the possibility of differentiating to mesoderm [49–52], endoderm [53, 54], and ectoderm [51, 55] under shear. Further, given the role of shear stress in fetal development [56–59], biomimetic flow may be beneficial to the function of the differentiated tissue. However, high levels of shear stress were detrimental in some instances of differentiation. For instance, Ting *et al* found continuous agitation on a rocker during cardiomyocyte differentiation resulted in very few cardiomyocytes; yet, intermittent agitation led to an ∼38% increase in differentiation efficiency compared to static culture [49]. Similarly, in a hepatic differentiation in microcarriers, agitation above 30 rpm caused cell detachment from the microcarriers during early stages of differentiation [54]. Therefore, parameters for differentiation in the bioreactor will need to be optimized for the desired tissue type in order to balance nutrient delivery/waste removal and maintenance of cell health.

The utility of *in situ* hiPSC proliferation can be further augmented as methods to induce multilineage differentiation progress [60–65]. Such methodology, ranging from varied scaffold material properties to altered cytokine or media compositions and genetically engineered hiPSCs, can be easily translated to bioprinting and the bioreactor set-up to obtain large numbers of different cell types. Differentiation to multiple tissue types would allow studies on the formation of relevant tissues during development, the crosstalk between different tissues in disease progression, or the potential effects of various therapeutics and doses on various organs. For instance, spatial differentiation to both pacemaker and chamber cardiomyocytes would greatly enhance drug screening studies, providing insight on both heart rhythm and pump function of a given therapy. Incorporation of fibroblasts, immune, or neural cells could also further enhance biomimetic functionality.

Similarly, many tissue engineering studies are now focusing on methods to incorporate blood vessels to increase nutrient supply and possible tissue thickness. Yet the inclusion of endothelial cells and other relevant cell types in our set-up may cause unwanted paracrine signaling that could affect the pluripotency or differentiation efficiency of the stem cells in the printed tissue [66]. Upon differentiation to the desired cell type, though, endothelial cells could be incorporated in a model such as ours, where perfusion could further enable mechanotransduction important for the endothelium [67, 68] and possibly induce angiogenesis [69]. The presence of blood vessels will allow for an increase in nutrient delivery to further develop the utility of engineered tissues.

Recent developments in the field of 3D bioprinting have greatly expanded the potential for creating complex, physiologically-relevant *in vitro* tissues. When such tissues are formed with human-derived cells, resulting models can provide enhanced studies of disease mechanisms, progression, and therapies, complementary to animal models. Here we take advantage of media perfusion and the high proliferative capacity of hiPSCs within a 3D-bioprinted chambered construct, towards the goal of generating a thick-tissue model. Overall, we anticipate that this ability to expand hiPSCs within such models will be important as the field moves forward in creating more complex tissues, particularly when combined with engineering strategies being developed. The formation of contiguous colonies of hiPSCs seen in this model will allow for *in situ* differentiation of various tissue types in order to generate tissues with increased functionality and consistency for reliable *in vitro* modeling.

## Data Availability

All data that support the findings of this study are included within the article (and any supplementary files).

## References

[1] Gudapati H, Dey M, Ozbolat I (2016). A comprehensive review on droplet-based bioprinting: past, present and future. Biomaterials.

[2] Skylar-Scott M A, Uzel S G M, Nam L L, Ahrens J H, Truby R L, Damaraju S, Lewis J A (2019). Biomanufacturing of organ-specific tissues with high cellular density and embedded vascular channels. Sci. Adv..

[3] Murata D, Arai K, Nakayama K (2020). Scaffold-free bio-3D printing using spheroids as ‘bio-inks’ for tissue (re-)construction and drug response tests. Adv. Healthcare Mater..

[4] Daly A C, Davidson M D, Burdick J A (2021). 3D bioprinting of high cell-density heterogeneous tissue models through spheroid fusion within self-healing. Nat. Commun..

[5] Kupfer M E (2020). *In situ* expansion, differentiation and electromechanical coupling of human cardiac muscle in a 3D bioprinted, chambered organoid. Circ. Res..

[6] Ouyang L, Yao R, Mao S, Chen X, Na J, Sun W (2015). Three-dimensional bioprinting of embryonic stem cells directs highly uniform embryoid body formation. Biofabrication.

[7] Faulkner-Jones A, Fyfe C, Cornelissen D-J, Gardner J, King J, Courtney A, Shu W (2015). Bioprinting of human pluripotent stem cells and their directed differentiation into hepatocyte-like cells for the generation of mini-livers in 3D. Biofabrication.

[8] Ouyang L, Yao R, Zhao Y, Sun W (2016). Effect of bioink properties on printability and cell viability for 3D bioplotting of embryonic stem cells. Biofabrication.

[9] Gu Q, Tomaskovic-Crook E, Wallace G G, Crook J M (2017). 3D bioprinting human induced pluripotent stem cell constructs for *in situ* cell proliferation and successive multilineage differentiation. Adv. Healthcare Mater..

[10] Li Y, Jiang X, Li L, Chen Z-N, Gao G, Yao R, Sun W (2018). 3D printing human induced pluripotent stem cells with novel hydroxypropyl chitin bioink: scalable expansion and uniform aggregation. Biofabrication.

[11] Hamid O A, Eltaher H M, Sottile V, Yang J (2021). 3D bioprinting of a stem cell-laden, multi-material tubular composite: an approach for spinal cord repair. Mater. Sci. Eng. C.

[12] Kerscher P, Turnbull I C, Hodge A J, Kim J, Seliktar D, Easley C J, Costa K D, Lipke E A (2016). Direct hydrogel encapsulation of pluripotent stem cells enables ontomimetic differentiation and growth of engineered human heart tissues. Biomaterials.

[13] Gilmozzi V (2021). Generation of hiPSC-derived functional dopaminergic neurons in alginate-based 3D culture. Front. Cell Dev. Biol..

[14] Gao L (2017). Myocardial tissue engineering with cells derived from human-induced pluripotent stem cells and a native-like, high-resolution, 3-dimensionally printed scaffold. Circ. Res..

[15] Radisic M, Malda J, Epping E, Geng W, Langer R, Vunjak-Novakovic G (2006). Oxygen gradients correlate with cell density and cell viability in engineered cardiac tissue. Biotechnol. Bioeng..

[16] Ahmed S, Chauhan V M, Ghaemmaghami A M, Aylott J W (2018). New generation of bioreactors that advance extracellular matrix modelling and tissue engineering. Biotechnol. Lett..

[17] Zhang J, Wehrle E, Rubert M, Müller R (2021). 3D bioprinting of human tissues: biofabrication, bioinks, and bioreactors. Int. J. Mol. Sci..

[18] Adamo L (2009). Biomechanical forces promote embryonic haematopoiesis. Nature.

[19] Yamamoto K, Sokabe T, Watabe T, Miyazono K, Yamashita J K, Obi S, Ohura N, Matsushita A, Kamiya A, Ando J (2005). Fluid shear stress induces differentiation of Flk-1-positive embryonic stem cells into vascular endothelial cells *in vitro*. Am. J. Physiol. Circ. Physiol..

[20] Ahsan T, Nerem R M (2010). Fluid shear stress promotes an endothelial-like phenotype during the early differentiation of embryonic stem cells. Tissue Eng. A.

[21] Greuel S, Freyer N, Hanci G, Böhme M, Miki T, Werner J, Schubert F, Sittinger M, Zeilinger K, Mandenius C-F (2019). Online measurement of oxygen enables continuous noninvasive evaluation of human-induced pluripotent stem cell (hiPSC) culture in a perfused 3D hollow-fiber bioreactor. J. Tissue Eng. Regen. Med..

[22] Fattahi P (2021). Core–shell hydrogel microcapsules enable formation of human pluripotent stem cell spheroids and their cultivation in a stirred bioreactor. Sci. Rep..

[23] Isidro I A (2021). Online monitoring of hiPSC expansion and hepatic differentiation in 3D culture by dielectric spectroscopy. Biotechnol. Bioeng..

[24] Borys B S (2021). Overcoming bioprocess bottlenecks in the large-scale expansion of high-quality hiPSC aggregates in vertical-wheel stirred suspension bioreactors. Stem Cell Res. Ther..

[25] Nogueira D E S, Rodrigues C A V, Carvalho M S, Miranda C C, Hashimura Y, Jung S, Lee B, Cabral J M S (2019). Strategies for the expansion of human induced pluripotent stem cells as aggregates in single-use Vertical-Wheel^TM^ bioreactors. J. Biol. Eng..

[26] Zhu W, Zhao M, Mattapally S, Chen S, Zhang J (2018). CCND2 overexpression enhances the regenerative potency of human induced pluripotent stem cell-derived cardiomyocytes: remuscularization of injured ventricle. Circ. Res..

[27] Lee A, Hudson A R, Shiwarski D J, Tashman J W, Hinton T J, Yerneni S, Bliley J M, Campbell P G, Feinberg A W (2019). 3D bioprinting of collagen to rebuild components of the human heart. Science.

[28] Figueroa C A, Vignon-Clementel I E, Jansen K E, Hughes T J R, Taylor C A (2006). A coupled momentum method for modeling blood flow in three-dimensional deformable arteries. Comput. Methods Appl. Mech. Eng..

[29] Ye J, Coulouris G, Zaretskaya I, Cutcutache I, Rozen S, Madden T L (2012). Primer-BLAST: a tool to design target-specific primers for polymerase chain reaction. BMC Bioinform..

[30] Kung E O, Les A S, Figueroa C A, Medina F, Arcaute K, Wicker R B, McConnell M V, Taylor C A (2011). *In vitro* validation of finite element analysis of blood flow in deformable models. Ann. Biomed. Eng..

[31] Bonfanti M, Franzetti G, Homer-Vanniasinkam S, Díaz-Zuccarini V, Balabani S (2020). A combined *in vivo,*
*in vitro,* in silico approach for patient-specific haemodynamic studies of aortic dissection. Ann. Biomed. Eng..

[32] Sargent C Y, Berguig G Y, Kinney M A, Hiatt L A, Carpenedo R L, Berson R E, McDevitt T C (2010). Hydrodynamic modulation of embryonic stem cell differentiation by rotary orbital suspension culture. Biotechnol. Bioeng..

[33] Wang Y, Chou B K, Dowey S, He C, Gerecht S, Cheng L (2013). Scalable expansion of human induced pluripotent stem cells in the defined xeno-free E8 medium under adherent and suspension culture conditions. Stem Cell Res..

[34] Ismadi M Z, Gupta P, Fouras A, Verma P, Jadhav S, Bellare J, Hourigan K (2014). Flow characterization of a spinner flask for induced pluripotent stem cell culture application. PLoS One.

[35] Farzaneh M (2021). Concise review; effects of antibiotics and antimycotics on the biological properties of human pluripotent and multipotent stem cells. Curr. Stem Cell Res. Ther..

[36] Carrier R L, Rupnick M, Langer R, Schoen F J, Freed L E, Vunjak-Novakovic G (2002). Perfusion improves tissue architecture of engineered cardiac muscle. Tissue Eng..

[37] Radisic M, Yang L, Boublik J, Cohen R J, Langer R, Freed L E, Vunjak-Novakovic G (2004). Medium perfusion enables engineering of compact and contractile cardiac tissue. Am. J. Physiol.—Heart Circ. Physiol..

[38] Dahlin R L, Meretoja V V, Ni M, Kasper F K, Mikos A G (2012). Design of a high-throughput flow perfusion bioreactor system for tissue engineering. Tissue Eng. C.

[39] Gabetti S (2022). An automated 3D-printed perfusion bioreactor combinable with pulsed electromagnetic field stimulators for bone tissue investigations. Sci. Rep..

[40] Yamada S, Yassin M A, Schwarz T, Mustafa K, Hansmann J (2022). Optimization and validation of a custom-designed perfusion bioreactor for bone tissue engineering: flow assessment and optimal culture environmental conditions. Front. Bioeng. Biotechnol..

[41] Nsiah B A, Ahsan T, Griffiths S, Cooke M, Nerem R M, McDevitt T C (2014). Fluid shear stress pre-conditioning promotes endothelial morphogenesis of embryonic stem cells within embryoid bodies. Tissue Eng. A.

[42] Blagovic K, Kim L Y, Voldman J (2011). Microfluidic perfusion for regulating diffusible signaling in stem cells. PLoS One.

[43] Nath S C, Day B, Harper L, Yee J, Hsu C Y-M, Larijani L, Rohani L, Duan N, Kallos M S, Rancourt D E (2021). Fluid shear stress promotes embryonic stem cell pluripotency via interplay between β-catenin and vinculin in bioreactor culture. Stem Cells.

[44] Wang Z, Oron E, Nelson B, Razis S, Ivanova N (2012). Distinct lineage specification roles for NANOG, OCT4, and SOX2 in human embryonic stem cells. Cell Stem Cell.

[45] Zhang X (2009). A role for NANOG in G1 to S transition in human embryonic stem cells through direct binding of CDK6 and CDC25A. J. Cell Biol..

[46] Watanabe K (2007). A ROCK inhibitor permits survival of dissociated human embryonic stem cells. Nat. Biotechnol..

[47] Kim K (2020). 3,2′-Dihydroxyflavone improves the proliferation and survival of human pluripotent stem cells and their differentiation into hematopoietic progenitor cells. J. Clin. Med..

[48] Pallardó F V, Markovic J, García J L, Viña J (2009). Role of nuclear glutathione as a key regulator of cell proliferation. Mol. Aspects Med..

[49] Ting S, Chen A, Reuveny S, Oh S (2014). An intermittent rocking platform for integrated expansion and differentiation of human pluripotent stem cells to cardiomyocytes in suspended microcarrier cultures. Stem Cell Res..

[50] Correia C (2014). Combining hypoxia and bioreactor hydrodynamics boosts induced pluripotent stem cell differentiation towards cardiomyocytes. Stem Cell Rev. Rep..

[51] Badenes S M, Fernandes T G, Cordeiro C S M, Boucher S, Kuninger D, Vemuri M C, Diogo M M, Cabral J M S (2016). Defined essential 8^TM^ medium and vitronectin efficiently support scalable xeno-free expansion of human induced pluripotent stem cells in stirred microcarrier culture systems. PLoS One.

[52] Ackermann M (2018). Bioreactor-based mass production of human iPSC-derived macrophages enables immunotherapies against bacterial airway infections. Nat. Commun..

[53] Lock L T, Tzanakakis E S (2009). Expansion and differentiation of human embryonic stem cells to endoderm progeny in a microcarrier stirred-suspension culture. Tissue Eng. A.

[54] Park Y, Chen Y, Ordovas L, Verfaillie C M (2014). Hepatic differentiation of human embryonic stem cells on microcarriers. J. Biotechnol..

[55] Qiu L, Lim Y M, Chen A K, Reuveny S, Oh S K W, Tan E K, Zeng L (2016). Microcarrier-expanded neural progenitor cells can survive, differentiate, and innervate host neurons better when transplanted as aggregates. Cell Transplant..

[56] Hove J R, Köster R W, Forouhar A S, Acevedo-Bolton G, Fraser S E, Gharib M (2003). Intracardiac fluid forces are an essential epigenetic factor for embryonic cardiogenesis. Nature.

[57] Courchaine K, Rykiel G, Rugonyi S (2018). Influence of blood flow on cardiac development. Prog. Biophys. Mol. Biol..

[58] Conrad L (2021). The biomechanical basis of biased epithelial tube elongation in lung and kidney development. Development.

[59] Lorenz L (2018). Mechanosensing by β1 integrin induces angiocrine signals for liver growth and survival. Nature.

[60] Ng W H (2022). Recapitulating human cardio-pulmonary co-development using simultaneous multilineage differentiation of pluripotent stem cells. eLife.

[61] Skylar-Scott M A, Huang J Y, Lu A, Ng A H M, Duenki T, Liu S, Nam L L, Damaraju S, Church G M, Lewis J A (2022). Orthogonally induced differentiation of stem cells for the programmatic patterning of vascularized organoids and bioprinted tissues. Nat. Biomed. Eng..

[62] Giacomelli E, Bellin M, Sala L, van Meer B J, Tertoolen L G J, Orlova V V, Mummery C L (2017). Three-dimensional cardiac microtissues composed of cardiomyocytes and endothelial cells co-differentiated from human pluripotent stem cells. Development.

[63] Wu F, Wu D, Ren Y, Huang Y, Feng B, Zhao N, Zhang T, Chen X, Chen S, Xu A (2019). Generation of hepatobiliary organoids from human induced pluripotent stem cells. J. Hepatol..

[64] Jin G, Floy M E, Simmons A D, Arthur M M, Palecek S P (2021). Spatial stem cell fate engineering via facile morphogen localization. Adv. Healthcare Mater..

[65] Kilian D, Cometta S, Bernhardt A, Taymour R, Golde J, Ahlfeld T, Emmermacher J, Gelinsky M, Lode A (2022). Core-shell bioprinting as a strategy to apply differentiation factors in a spatially defined manner inside osteochondral tissue substitutes. Biofabrication.

[66] Freyer N, Greuel S, Knöspel F, Strahl N, Amini L, Jacobs F, Monshouwer M, Zeilinger K (2017). Effects of co-culture media on hepatic differentiation of hiPSC with or without HUVEC co-culture. Int. J. Mol. Sci..

[67] Davies P F (2009). Hemodynamic shear stress and the endothelium in cardiovascular pathophysiology. Nat. Clin. Pract. Cardiovasc. Med..

[68] Kinstlinger I S, Calderon G A, Royse M K, Means A K, Grigoryan B, Miller J S (2021). Perfusion and endothelialization of engineered tissues with patterned vascular networks. Nat. Protocols.

[69] Galie P A, Nguyen D H T, Choi C K, Cohen D M, Janmey P A, Chen C S (2014). Fluid shear stress threshold regulates angiogenic sprouting. Proc. Natl Acad. Sci. USA.

